# Polyols from Microwave Liquefied Bagasse and Its Application to Rigid Polyurethane Foam

**DOI:** 10.3390/ma8125472

**Published:** 2015-12-08

**Authors:** Jiulong Xie, Xianglin Zhai, Chung Yun Hse, Todd F. Shupe, Hui Pan

**Affiliations:** 1 School of Renewable Natural Resources, Louisiana State University Agricultural Center, Baton Rouge, LA 70803, USA; jxie6@lsu.edu (J.X.); xzhai1@lsu.edu (X.Z.); tshupe@agcenter.lsu.edu (T.F.S.); 2 Southern Research Station, USDA Forest Service, Pineville, LA 71360, USA; chse@fs.fed.us; 3 College of Chemical Engineering, Nanjing Forestry University, Nanjing 210037, China

**Keywords:** liquefaction, bagasse, microwave, polyhydric alcohol, polyurethane foams

## Abstract

Bagasse flour (BF) was liquefied using bi-component polyhydric alcohol (PA) as a solvent and phosphoric acid as a catalyst in a microwave reactor. The effect of BF to solvent ratio and reaction temperatures on the liquefaction extent and characteristics of liquefied products were evaluated. The results revealed that almost 75% of the raw bagasse was converted into liquid products within 9 min at 150 °C with a BF to solvent ratio of 1/4. The hydroxyl and acid values of the liquefied bagasse (LB) varied with the liquefied conditions. High reaction temperature combining with low BF to solvent ratio resulted in a low hydroxyl number for the LB. The molecular weight and polydispersity of the LB from reactions of 150 °C was lower compared to that from 125 °C. Rigid polyurethane (PU) foams were prepared from LB and methylene diphenyl diisocyanate (MDI), and the structural, mechanical and thermal properties of the PU foam were evaluated. The PU foams prepared using the LB from high reaction temperature showed better physical and mechanical performance in comparison to those from low reaction temperature. The amount of PA in the LB has the ability of increasing thermal stability of LB-PU foams. The results in this study may provide fundamental information on integrated utilizations of sugarcane bagasse via microwave liquefaction process.

## 1. Introduction

In recent years, there has been an increased desire for more effective utilization of lignocellulosic biomass waste due to its significant potential to enhance environmental stewardship and aid economic development. Lignocellulosic biomass composed of cellulose, hemicellulose and lignin is a valuable and worldwide-accessible bioresource which can provide alternative chemicals via proper conversion processes. Recent achievements in biomass thermochemical conversion techniques have stimulated great interest in the integrated utilizations of lignocellulosic biomass for the production of hydroxyl-rich biopolyols. Various biomass conversion technologies have received increasing attention. Thermochemical methods such as pyrolysis and liquefaction have great potential to produce biofuels and valuable bio-chemicals [1,2]. Studies have shown that liquefaction provides an efficient pathway to convert solid biomass into liquid products [3,4].

Sugarcane is an important crop cultivated around the world and plays a vital role in aural-based agricultural economics. The major by-product of the sugarcane industry is sugarcane bagasse (SB) [5]. Usually, SB is used as a source of heat and electricity in sugar producing mills. With the development of lignocellulosic biomass utilization technologies, SB was also applied in the preparation of oriented strand board as well as the feedstock for the isolation of cellulosic fibers [6,7,8]. Recently, many previous studies have explored the potential of liquefaction sugarcane bagasse using traditional heating sources with long liquefaction times and low energy efficiency [9,10,11,12,13]. Sugarcane bagasse is mainly composed of approximately 50% cellulose, 25% hemicellulose and 22% lignin [14]. The components of sugarcane bagasse are known to have high functional hydroxyl groups because of the high cellulose and hemicellulose contents. Thus, liquefied bagasse (LB) is considered an excellent raw material for bio-based polyols, which have been used to manufacture epoxy resins [15]. These studies have shown the potential of developing bio-based products from underutilized bagasse. These studies have also indicated the need for further improvement in liquefaction technology such as lower acid catalyst content, shorter reaction time, lower reaction temperature and lower organic solvent ratio before an economically viable conversion technology can be realized.

The application of microwave heating in lignocellulosic biomass liquefaction was first applied in wood liquefaction [16]. Microwave irradiation can directly couple microwave energy with the molecules that are present in the reaction mixture with dipolar polarization and ionic conduction. The main advantage of microwave over conventional heating sources is that the irradiation penetrates and simultaneously heats the materials at the molecular level and therefore, reduces reaction times from hours to minutes [17,18,19,20]. Microwave energy has been applied in the liquefaction of various lignocellulosic biomasses such as pine [21], bamboo [22] and agricultural crop residues [23]. However, no publications have reported on the microwave liquefaction of sugarcane bagasse.

Moreover, Ethylene glycol, the monomer of polyethylene glycol (PEG), has a loss tangent (tan δ) value of 1.35, which is among the highest tan δ value of common solvents [21]. The higher the tan δ value of a solvent, the better absorption and more efficient heating capability the solvent has under microwave irradiation. Therefore, liquefaction using microwave energy as the heating source and PEG as a solvent has great potential to reduce reaction time and advance the commercialization of this process. The application of microwave energy to biomass liquefaction offers not only faster heating and energy efficiency but also space savings and precise process control [23].

Polyurethane foams are versatile engineering materials and have been successfully used in a variety of applications (e.g., automotive industry, refrigerators, insulating panels, construction) [24]. Commercially, polyurethane (PU) foam is synthesized by the reaction of polyol and dissocyanate with a combination of a blowing agent, catalyst, and surfactant. Currently, polyols and isocyanate used for PU foam production are mainly petroleum-derived. With increasing concern for the depletion of fossil fuels, global warming and other environmental impacts from petroleum-based products, the replacements of petroleum-based polyols with sustainable bio-polyols from renewable biomass for the production of PU foams have been receiving increasing attention.

Biopolyols obtained by liquefaction have high hydroxyl functionalities and great potential in the production of PU foams [25]. A large variety of lignocellulosic biomass such as pine wood [21], bamboo [22,26], wheat straw [27] and soybean straw [28] have been liquefied into liquid polyols for the preparation of PU foams. The incorporation of biomass components in polymeric compositions of PU foams can reduce manufacturing costs and provide a certain degree of biodegradability. Thus, the goal of this study was to determine the potential of microwave-assisted liquefaction of sugarcane bagasse as the raw material for rigid polyurethane foam. The specific objectives were to determine the effects of bagasse flour (BF)/poly alcohol (PA) ratio (w/w) on the properties of LB polyols and PU foams.

## 2. Results and Discussion

### 2.1. Liquefaction and Characteristics of LB

**Figure 1:**
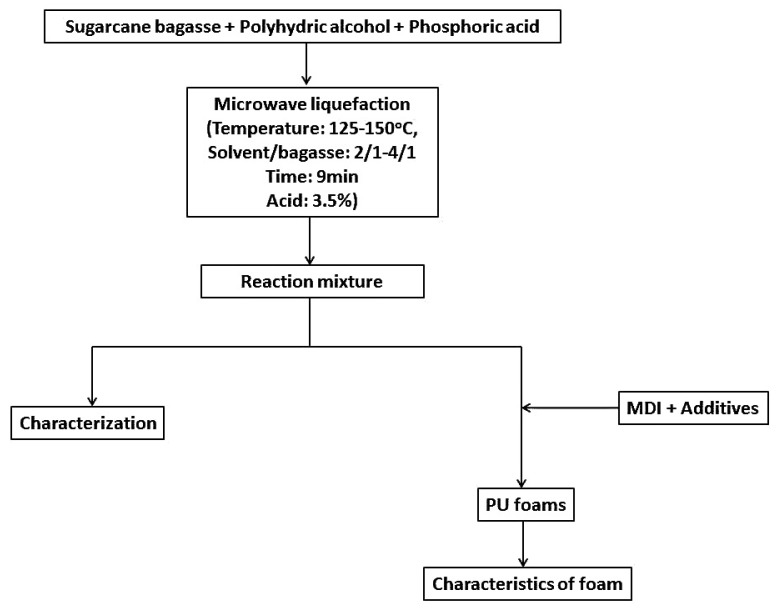
Experimental scheme of microwave liquefaction sugarcane bagasse.

**Figure 2:**
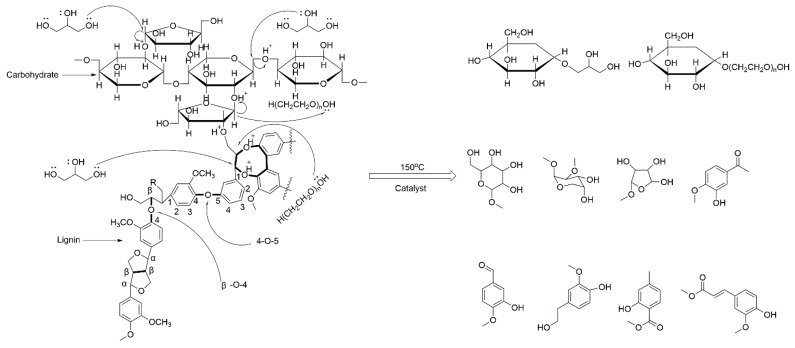
Proposed microwave liquefaction mechanism of sugarcane bagasse.

Figure 1 shows the experimental scheme. Sugarcane bagasse was liquefied at different liquefaction conditions using microwave energy. The liquefied products were characterized and applied in the preparation of polyurethane foams. The proposed reaction mechanism is presented in Figure 2. According to our previous results [3,29], the liquefaction of sugarcane bagasse was proposed to be the cleavage of several specific linkages in the bagasse, such as glycosidic bonds that are dominate linkages between the sugar units of cellulose and hemicellulose, and dominate linkages in lignin. The cleavage of glycosidic bonds in cellulose and ethers bonds in hemicellulose resulted in the production of carbon six and carbon six sugar derivatives, and the cleavage of the β-O-4, 4-O-5, and dibenzodioxocin linkages in lignin resulted in the aromatic products. Since PEG and glycerol used as the co-solvent in the liquefaction system, glycoside may also be produced by glycolysis.

**Figure 3:**
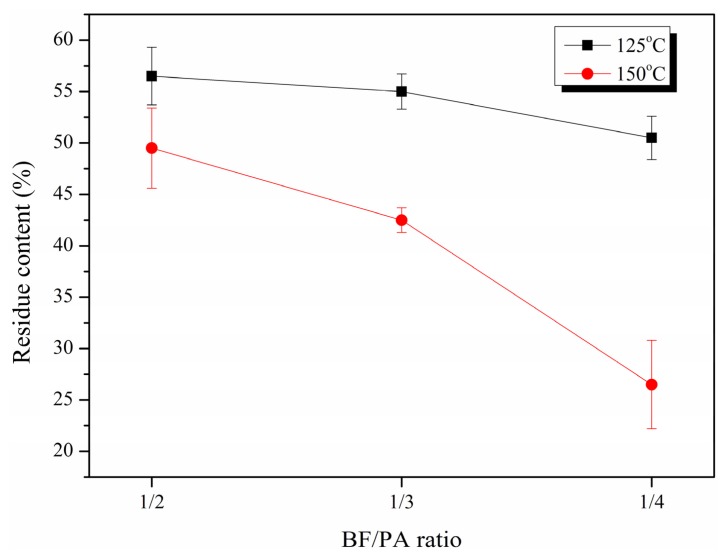
Effect of Bagasse flour (BF)/polyhydric alcohol (PA) ratio on the residue content of bagasse under two temperature levels.

The content of the liquefied residue (unreacted sugarcane bagasse) was defined as the percent of the weight of the dry residue to that of the dry raw material charged. The residue content was used as an indicator of the liquefaction extent. Figure 3 shows the changes of the residue content as a function of the BF/PA ratio at different reaction temperatures. As expected, the residue content decreased as the BF/PA ratio decreased, and the liquefaction efficiency was enhanced with an increase of liquefaction temperature. However, liquefaction temperature interacted with BF/PA ratio to impact the residue content. At a high BF/PA ratio (*i.e.*, 1/2), the temperature had little effect on residue content as BF/PA ratio decreased to 1/3, the residue content decreased significantly for both liquefaction temperatures. The decrease in residue content leveled off with a decrease in the BF/PA ratio at 125 °C; while the residue content gradually decreased to the lowest residue content at 150 °C. It is evident that higher temperature resulted in less residue content for all BF/PA ratios. As the amount of PA in the mixture increased, the difference between the residue content in the same BF/PA ratio under the two temperatures became gradually notable. Thus, it could be concluded that the effect of temperature on the liquefaction rate of LB is greatly dependent on the BF/PA ratio.

Hydroxyl value of the polyols is an important parameter that needs to be monitored during polyols and polyurethane foam production. The hydroxyl value of the polyols was determined as 426–505 mg KOH/g. The synthesized polyol from SB in this study had a higher hydroxyl value than the liquefaction of bamboo as reported in the literature indicating that the biopolyols synthesized in this study had good reactivity with isocyanate [26].

**Figure 4:**
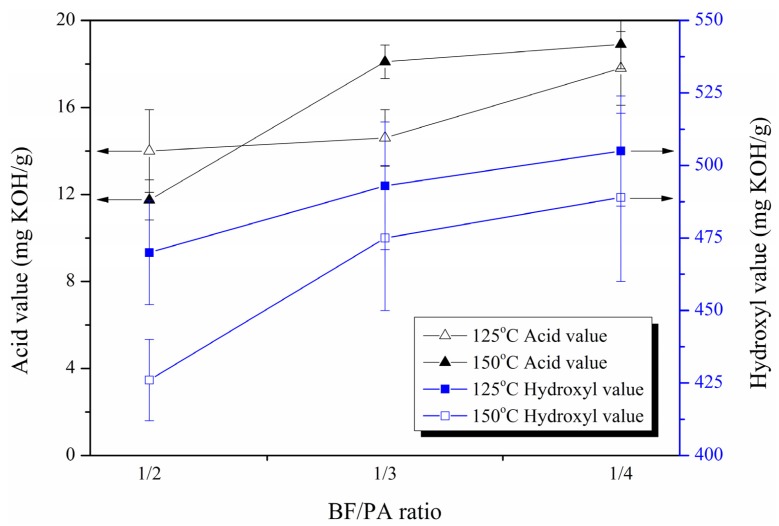
Effect of BF/PA ratio on the acid and hydroxyl values of liquefied bagasse polyols prepared under two temperature levels.

The effect of BF/PA ratio on acid value and hydroxyl value of the liquefied products (polyols) from 125 and 150 °C are shown in Figure 4. It was observed that with an increase in the PA amount in the mixture, the hydroxyl value of the polyols gradually increased. Moreover, high temperature contributed to smaller hydroxyl values, *i.e.*, the hydroxyl value of the polyols prepared at 125 °C was higher than that of the polyols prepared at 150 °C. This indicates that although the PA in the mixture can provide the hydroxyl group of the polyols, a loss of hydroxyl groups occurred during the liquefaction reaction which could be largely attributed to the alcoholysis reaction of bagasse in PA and to the formation of ethers. The oxidation and recondensation reactions among the liquefaction solvents and the decomposed bagasse components could also take place during the liquefaction to consume hydroxyl groups and influence the hydroxyl value. With decreasing BF/PA ratio, the hydroxyl value slightly increased. This result is in accordance with the finding of the work on the liquefaction of soybean straw [28]. The higher hydroxyl value for the polyols from the reaction with high solvent loading may be due to the higher biomass conversion and that extra solvent in the reaction mixture avoided the recondensation reactions.

The LB bagasse was acidic due to the acid catalyst used in the liquefaction system as well as the acid substance decomposed from the wood components, mainly cellulose and hemicellulose [21]. The acid value of the LB was in the range of 11.8–18.9 mg KOH/g. The increase of acid value could be attributed to the increase of the PA, which contained 3.5 wt.% H_3_PO_4_, and also to the increase of acidic substances produced with the decomposition of bagasse components and the oxidation of alcohols as the liquefaction proceeded [30]. This provided further evidence that the BF/PA ratio and temperature can both enhance the liquefaction rate.

The molecular weight and the polydispersity of the liquefied product are presented in Table 1. It is observed that, along with the increase of the PA amount in the mixture, the weight-average molecular weight (Mw) and polydispersity (Mw/Mn) leveled off. There was a slight decrease of Mw and Mw/Mn when the BF/PA ratio decreased from 1/2 to 1/3. The statistical analysis did not reveal any significant differences. It was also seen that the Mw of LB polyols acquired under higher temperature (150 °C) was generally smaller than that of LB polyols acquired under lower temperature (125 °C). This may be because microwave radiation under higher temperature shows a stronger ability to break chemical bonds such as dominating β-O-4 linkages in lignin and glycosidic bond in cellulose.

**Table 1:** Effects of BF/PA ratio on Mw and Mw/Mn of liquefied bagasse polyols prepared under two temperature levels.

BF/PA Ratio	Temperature (°C)	Mw	Mn
1/2	125	790 ± 83 ^a^	1.86 ± 0.14
	150	675 ± 57	1.76 ± 0.09
1/3	125	742 ± 64	1.77 ± 0.10
	150	600 ± 41	1.75 ± 0.13
1/4	125	855 ± 73	1.91 ± 0.17
	150	740 ± 36	1.74 ± 0.06

^a^ Mean ± standard deviation of three replicates.

**Figure 5:**
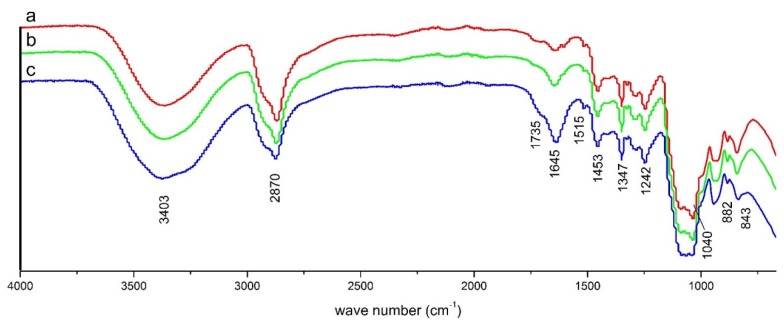
FTIR spectra of acetone soluble fraction of liquefied bagasse (LB) prepared at different BF/PA ratios (**a**. 1/2; **b**. 1/3; **c**. 1/4) in 150 °C.

Figure 5 shows the FTIR spectra of the acetone-soluble fraction of LB. A broad peak around 3400 cm^−1^ represents the OH groups either from cellulose or from unreacted PA. The peak around 2870 cm^−1^ represents the C-H symmetric stretching in aliphatic methyl. A shoulder at 1735 cm^−1^ is primarily due to the carbonyl stretch in unconjugated ketone, ester or carboxylic groups in hemicelluloses [31,32]. From the growing shoulder of the 1735 cm^−1^ peak, it can be inferred that hemicellulose is peeled off from adjacent lignin or cellulose into solution [33,34]. As the BF/PA ratio decreases, the absorbance band of hemicellulose was merged into the prominent peak at 1645 cm^−1^ which is associated with the adsorbed water [35]. The increase in the intensity of this peak in the acetone-soluble fraction of LB with respect to the increase in PA loading is due both to the removal of hemicellulose from BF [36] and the increase of the PA content in LB.

The spectra also showed that the acetone soluble fraction of LB had characteristic bands of benzene rings (1515 cm^−1^, 1453 cm^−1^, 1242 cm^−1^), especially the bands of syringyl rings (1347 cm^−1^, 1242 cm^−1^), which indicated that the acetone-soluble fraction contained the derivatives from lignin components. These bands showed no significant difference in the spectra of the polyols from different BF/PA ratio reactions revealing that the BF/PA ratio had no significant influence on the structure of the phenolic components in the polyols. This is mainly because lignin in the SB could easily undergo decomposition at the initial reaction stage in the microwave liquefaction system [37] and further increasing the PA composition would not affect the lignin depolymerization mechanism.

There were also weak bands of plane deviational vibration of –OH in carboxylic groups at 1409 cm^−1^ and out-of-plane deforming vibration of O-H in carboxylic groups at 882 cm^−1^. The carboxylic acids could be the degradation products of cellulose or hemicelluloses. The intensive band at 1040 cm^−1^ arises from the aromatic C-H in-plan deformation for guaiacyl type in lignin. The peak became weaker with an increase in PA. This is because the concentration of guaiacyl unit decreases in the fraction. Aromatic C-H out of bending occurs at 843 cm^−1^ [38]. The spectra of acetone soluble fraction of LBs prepared in 125 °C were similar to those prepared at 150 °C.

### 2.2. Characteristics of PU Foam

**Figure 6:**
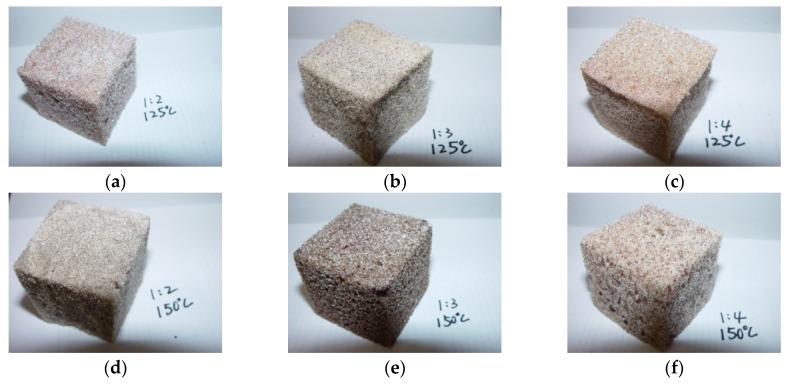
Photographs of the prepared polyurethane foams. Liquefied polyols were obtained at (**a**) BF/PA = 1/2, liquefaction temperature = 125 °C; (**b**) BF/PA = 1/3, liquefaction temperature = 125 °C; (**c**) BF/PA = 1/4, liquefaction temperature = 125 °C; (**d**) BF/PA = 1/2, liquefaction temperature = 150 °C; (**e**) BF/PA = 1/3, liquefaction temperature = 150 °C; (**f**) BF/PA = 1/4, liquefaction temperature = 150 °C.

Polyurethane (PU) foams were prepared from LB polyols and isocyanate with catalysts and additives. The LB could be used as the polyol component because it comprises the skeleton and active functional groups of polyols as indicated by the high hydroxyl number. Since the LB was a by-product of the sugar industry, foam developed using LB was thought to be sustainable and its cost was speculated to be lower than the petro-polyol. The LBs from different BF/PA ratios at different reaction temperatures were used for the PU foam synthesis in order to verify their capability for foam production. All the PU foams synthesized in this study were rigid type and the foam became darker in color with the addition of LB polyols (Figure 6). The hydroxyl values of the LB polyols were generally less than that of PA. Therefore, the usage of methylene diphenyl diisocyanate (MDI) in preparing PU foams based on LB polyols was less than that based on PA.

The density and physical properties of the PU foam samples prepared from liquefied bagasse polyols are shown in Table 2. The density increased as the PA content increased under both temperature levels. Also, higher temperature (150 °C) resulted in higher PU foam densities (in the range of 0.032–0.043 g/cm^3^). In contrast, the density of the PU foams prepared under lower temperature (125 °C) was lower (in the range of 0.031–0.037 g/cm^3^). The results indicate that the density of PU foams could be adjusted by controlling certain liquefaction conditions. As shown in Table 2 at 125 °C, the compressive strength (CS) and the modulus of elasticity (MOE) of the foams increased from 0.190 to 0.340 MPa and from 1.1 to 3.02 MPa, respectively, as the BF/PA ratio decreased from 1/2 to 1/4. When increasing the reaction temperature to 150 °C and keeping the other conditions the same, the CS and the MOE of the foams increased from 0.32 to 0.48 MPa and from 2.0 to 5.1 MPa, respectively, with a decrease in BF/PA ratio. Clearly, higher reaction temperature favors more robust foam.

**Table 2:** Density of polyurethane foams with respect to temperature and LB/PA ratio.

BF/PA Ratio	Temperature (°C)	Density (g/cm^−3^)	CS ^a^ (Mpa)	MOE ^b^ (Mpa)
1/2	125	0.031 ± 0.003 ^c^	0.19 ± 0.022	1.1 ± 0.045
	150	0.032 ± 0.000	0.32 ± 0.017	2.0 ± 0.020
1/3	125	0.032 ± 0.001	0.28 ± 0.027	1.2 ± 0.039
	150	0.035 ± 0.002	0.33 ± 0.007	3.4 ± 0.061
1/4	125	0.037 ± 0.005	0.34 ± 0.076	3.1 ± 0.033
	150	0.043 ± 0.007	0.48 ± 0.051	5.1 ± 0.083

^a^ compressive strength of the foams; ^b^ modulus of elasticity of the foams; ^c^ Mean ± standard derivation of three replicates.

These changes may be due to the interaction of the hydroxyl values of LB polyols and the liquefied bagasse residue in the polyols. As the PA content in the liquefied mixture increased, the hydroxyl value of the LB polyols also increased (Figure 4). The CS of the foam was influenced by the amount of PA in the liquefied mixture via the change of the hydroxyl value of the polyols. In terms of the interrelationships, a higher degree of cross-linking of the foam was due to higher hydroxyl values of the polyols. This is the reason why the CS and MOE of PU foams increased as the PA content of the liquefied polyols increased. However, the liquefied temperature had an inverse effect on the CS as that of the hydroxyl values of the polyols. To this point, the change of the CS followed that of the densities. This indicates that the impact of density was greater than the impact of liquefied temperature on the CS of PU foam.

Furthermore, due to the increase of the extent of bagasse liquefaction, the morphological properties of the residue were more homogeneous and had a higher surface area. This allows for better adhesion between the bagasse residue and PU. Hence, better CS can be acquired. In addition, it should be noted that at a fixed ratio of isocyanate index with the different weight percentage of bagasse residue content (Figure 3), unreacted components during liquefaction could not provide effective strength for the foams. The higher the bagasse residue content, the lower the economic cost for the PU foams, but the mechanical properties were poor. Therefore, the balance point between the economic cost and mechanical properties should be reached based on a practical application. Based on the results in the present study, in terms of obtaining PU foams with high physical and mechanical properties, the preferred liquefaction conditions should be at 150 °C, 9 min with a BF/PA ratio of 1/4. The resilience rates of the PU foams based on LB polyols from 1/2, 1/3 and 1/4 BF/PA ratio at 150 °C were 82.73, 83.27 and 82.49%, respectively; no significant difference was found.

**Figure 7:**
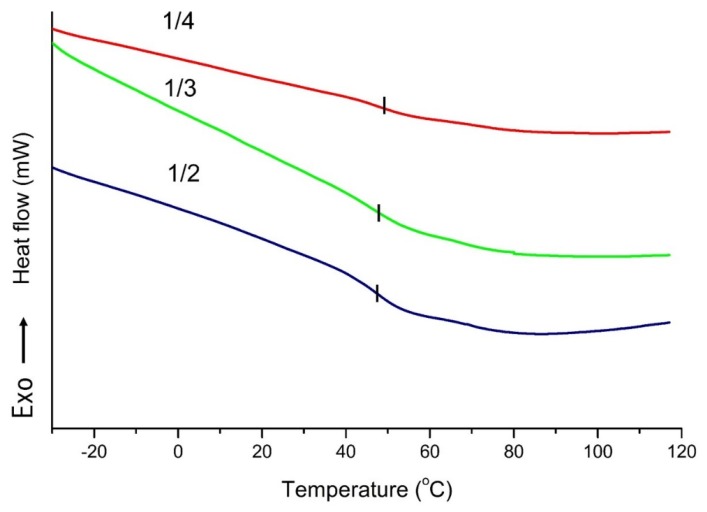
Differential scanning calorimetry (DSC) scans of polyurethane (PU) foams prepared with polyols form different LB/PA ratios at 150 °C.

Figure 7 shows the differential scanning calorimetry (DSC) curves of PU foams based on LB polyols prepared at 150 °C, and a slight change was observed. It was expected that the PU foams with greater cross-linking densities needed more thermal energy to initiate chain movements. As discussed previously, the hydroxyl values of the polyols increased with the PA content in the liquefied mixture. Higher hydroxyl values of the polyols were conducive to a higher degree of cross-linking in molecular chains of PU [39]. Hence, the change of the *T_g_* follows the change of the PA amount in the liquefied mixture.

**Figure 8:**
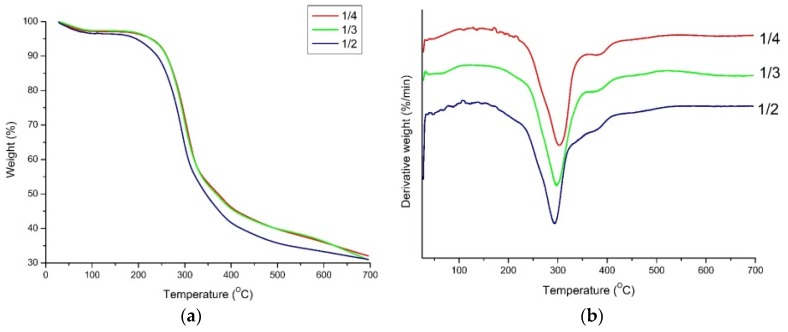
Thermogravimetry (**a**) and differential thermogravimetry; (**b**) curves of PU foams prepared with liquefied bagasse with a BF/PA ratio of 1/2, 1/3, and 1/4 at 150 °C.

Thermal decomposition behaviors of PU foams from liquefied bagasse at 150 °C are presented in Figure 8. From the thermogravimetry (TG) curves, it was observed that foams from BF/PA ratio of 1/4 was the most thermally stable followed by 1/3. PU foam from BF/PA ratio of 1/2 had the lowest thermal stability. This indicates that crosslinking density of foams increased with an increase in PA amount. With an increase in PA amount, the extent of liquefaction increased, which can be one of the reasons for the increase in crosslinking density (Figure 3). In the process of liquefaction when using H_3_PO_4_ as a catalyst and the corresponding increase of its amount with an increase of PA (as discussed above), it was possible that the cellulose in the insoluble residues was changed into cellulose phosphate, which has the characteristic of thermal durability. Moreover, due to liquefaction, more active OH groups on BF were possibly exposed in the polyols prepared with more PA. Thus, the insoluble residues created in the greater PA liquefaction process could act as a more efficient cross-linking agent [40]. These two factors could induce the direction of thermal stabilities of LB-PU foams.

From the curves of differential thermogravimetry (DTG), it is evident that the decomposition mainly occurred in three successive stages above 100 °C. The loss below 100 °C was attributed to the evaporation of moisture content and the release of volatile components. The initial decomposition started at 177.32 °C, and the rate of weight loss began to gradually increase to a maximum at about 302 °C. This suggests that decomposition started at the urethane bond. Urethanes are known to be relatively thermally unstable materials, primarily due to the presence of urethane bond decomposition which occurs somewhere between 150 and 220 °C depending on type of the substituents on the isocyanate and polyol side [41]. Moreover, because of the existence of liquefied residue in the samples, the degradation of bagasse constitutions, *i.e.*, hemicellulose and cellulose are at this temperature range [39,40]. The second stage, a shoulder in the DTG curve around 361.51 °C, could result in the degradation of isocyanate, which did not react with polyol or water (see Figure 5. peak at 2270 cm^−1^). The third stage (415.53–565.18 °C) largely attributed to the degradation of lignin and char residue from the second stage [38]. In conclusion, the amount of PA in the LB has the ability of increasing thermal stability of LB-PU foams.

## 3. Experimental Section

### 3.1. Materials and Chemicals

Bagasse was obtained from a local sugarcane processing mill near Baton Rouge, Louisiana, USA. Particles in the size range from 0.90 mm to 1.1 mm were used as the raw material for liquefaction experiments. The bagasse particles were dried at 105 °C for 24 h before use. The polyhydric alcohol (PA) used in the reactions was a mixture of polyethylene glycol (PEG, average molecular weight 400) and glycerol at a fixed weight ratio of 70/30. Phosphoric acid was used as the acidic catalyst. These chemicals were purchased from a commercial source. Methylene diphenyl diisocyanate (MDI; MR-100, Huntsman Industries Ltd., Alvin, TX, USA (NCO group content 30.03%)) and silicone surfactant (SH193; Toray Dow Corning Silicone Ltd., Tokyo, Japan) were used for the preparation of polyurethane foam. Deionized water was used as the blowing agent. All chemicals used were of reagent grade.

### 3.2. Liquefaction of Bagasse

The reactions were carried out in a Milestone MEGA 1200 laboratory microwave oven equipped with a temperature sensor that could be directly inserted into a sealed 100 mL PTFE (microwave transparent) reaction vessel. Oven-dried BF was pre-mixed with the solvent at a mass ratio of 1:2, 1:3, and 1:4. Phosphoric acid was added in the amount of 3.5 wt.% of the solvent. Each vessel was filled with 15 ± 0.04 g of mixture. Eight vessels of mixture were pulse radiated at a microwave frequency of 2.45 GHz and maximum power of 1000 W for 9 min. at two levels of temperature (125, 150 °C). The liquefaction temperature was monitored by a fiber-optic probe that was immersed at the center of a vessel. After radiation, the samples were cooled to room temperature. The resultant LB, including all of the components in the eight vessels, was collected for subsequent application and analysis.

Ten grams of LB was dissolved in 200 mL of dioxane/water binary solvent (4/1, *v*/*v*), stirred for more than 4 h, and then the solution was vacuum-filtrated through glass filter paper. The solid residues were dried in an oven at 105 °C to a constant weight, and the residue content was calculated based on the following equation:

(1) $$Residue\;\;content=\frac{W_{2}-W_{3}}{W_{1}}\times 100$$

where *W* is the weight of the LB polyols, and *W*_2_ and *W*_3_ are the dry weights of the filter paper with the residues and the weights of the filter paper, respectively.

### 3.3. Preparation of LB-PU Foam

The foams were prepared by a one-step method. A mixture of 25 g LB, 0.5 g deionized water, 0.25 g silicone and 0.25 g dibutyltine dilaurate was thoroughly premixed in a 150 mL paper cup with a mechanical stirrer for 1 min. Afterwards, a calculated amount of MDI (based on isocyanate index 110) was added to the pre-mixture. Then, the combination was stirred at room temperature with a high-speed agitator at a stirring speed of 3600 rpm for 3 min. The resultant mixture was immediately poured into an open cylindrical mold with a diameter of 20 cm and a height of 30 cm and allowed to freely rise at room conditions. The resulting foams were allowed to cure at room conditions for 1 h before being removed from the mold. The properties of the foams were measured after they were further conditioned at room conditions for two days. Three foams were prepared and tested for each liquefaction condition. The samples were prepared at an isocyanate index of 110. The isocyanate index ratio was determined as follows:

(2) $$Isocyanate\;\;index=\frac{M_{MDI}\times W_{MDI3}}{M_{LB}\times W_{LB}+W_{WATER}\times \left(2/18\right)}\times 100$$

where *MMDI* is the number of moles of isocyanate groups per gram of *MDI*, *MLB* is the number of moles of hydroxyl groups per gram of liquefied bagasse polyols; *WMDI*, *WLB* and *WWATER* are the weights (g) of MDI, LB polyols and water, respectively.

### 3.4. Characteristics of LB

#### 3.4.1. Acid Number

A method described by Kurimoto *et al.* was employed to determine the acid value [42]. A mixture of 10 g of LB and 200 mL of acetone/water (4:1, *v*/*v*) was titrated with 0.1 N potassium hydroxide standard solution to the equivalence point using a pH meter. The acid value (mgKOH/g) of the sample was calculated using the following equation:

(3) $$Acid\;\;value=\frac{(C-B)\times N\times 56.1}{W}$$

where *C* is the titration volume of the sodium hydroxide solution at the equivalence point (mL), *B* is the sodium hydroxide solution, and *W* is the weight of the LB sample (g).

#### 3.4.2. Hydroxyl Number

The method detailed by Kurimoto *et al.* (1999) was also used to determine the hydroxyl value [41]. LB (1 g) was esterified with phthalate reagent (25 mL) for 1 h at 110 °C followed by addition of 20 mL pyridine/deionized water (1:1, *v*/*v*), and then the mixture was titrated with 0.1 N potassium hydroxide standard solution to the equivalence point. The phthalate reagent was a mixture of 150 g phthalic anhydride. The hydroxyl value (mg KOH/g) was calculated by the following equation:

(4) $$Hydroxyl\;\;value=\frac{(B-A)\times N\times 56.1}{W}+\;acid\;value$$

where *A* is the volume of the potassium hydroxide solution required for titration of a LB sample (mL), B is the volume of blank solution (mL), *N* is the normality of the sodium hydroxide solution, and *W* is the weight of LB sample (g).

#### 3.4.3. Gel Permeation Chromatography (GPC) Analysis

Measurement of molecular weight by the GPC measurements were performed on a Waters-Wyatt GPC system equipped with multi-angle laser light scattering and differential refraction index detectors. Two Jordi Flash Gel Mixed Bed columns (250 × 10 mm) were used in series. Tests were conducted at ambient temperature using tetrahydrofuran (THF) as the mobile phase at a flow rate of 1.0 mL/min.

### 3.5. Characteristics of Polyurethane Foams

#### 3.5.1. Differential Scanning Calorimeter (DSC)

The glass transition temperature (*T_g_*) was measured on a Q20 differential scanning calorimeter, using a sealed aluminum capsule. The foam samples approximately 6 mg were investigated in nitrogen atmosphere from −40 to 120 °C, at a heating rate of 10 °C/min and a nitrogen flow rate of 50 mL/min. After 20 °C/min. programmed cooling, the samples were reheated at the same heating rate. The *T_g_* values were determined by analyzing the DSC curves from the second runs.

#### 3.5.2. Thermal Gravimetric Analysis (TGA)

TGA of the LB-PU foams samples was carried out using a TA Instrument Q 50. Samples with weights of approximately 5 mg were tested in each experiment. Nitrogen was used as the carrier gas at a flow rate of 40 mL/min. The heating rate was 10 °C/min. from room temperature to 700 °C. Curves of the foam samples were generated by a TA Instruments Universal Analysis 2000 version 4.7A Build 4.7.0.2.

#### 3.5.3. Mechanical Properties of Foams

Measurements of the compressibility of the foams were based on Japanese Industrial Standard [43]. The foams were cut into 50 mm^3^ specimens. The specimens were conditioned for two days at 23 °C and 50% relative humidity and then were measured and weighed to determine sample density. The compressive properties of the foams were then measured by means of a universal testing machine (INSTRON-4411). The measurements were made in the direction perpendicular to the foam rise direction at a constant crosshead speed of 5 mm/min. The compressive strength (CS) and modulus of elasticity (MOE) of the foams were determined at 10% stress and 25% strain. For each compression run, three pieces of each sample were used.

Resilience rate refers to the ability of a material to recover its original shape after it has been deformed. It is a measure of elastic characteristics and resilience and was determined using the same INSTRON universal testing machine as described above. A spring index of 100% indicates that the object behaves like a perfect elastic body. When the compression or tension force is released, the object will recover all of the deformation to its original dimensions.

A 60-mm diameter cylindrical probe was used to compress the samples to achieve a deformation of 80% of their original dimension at a loading rate of 30 mm/min. For each compression run, three pieces of each sample were used. Resilience rate was calculated by dividing the thickness after withdrawal of the compressive force by the initial thickness.

### 3.6. Data Analysis

The effects of liquefied temperature and BF and PA ratio on LB polyols properties and foam properties were evaluated by analysis of variance at the 0.05 level of probability. Significant effects were further characterized by the Duncan test.

## 4. Conclusions

Bagasse was subjected to a microwave-assisted liquefaction system. The influence of BF/PA ratio as well as the reaction temperature on liquefaction yield and characteristics of liquefied products was evaluated. With a decrease in BF/PA ratio, the residue content decreased. The BF/PA ratio and temperature had co-effects on residue content. Both the hydroxyl and acid values inversely increased with the BF/PA ratio at two different temperature levels. The Mw and Mw/Mn first increased and then leveled off with a decrease in BF/PA ratio. Polyurethane foams were prepared from the obtained liquefied polyols. The results revealed that with the addition of PA in the liquefaction mixer, the density, mechanical properties (compressive strength and MOE), glass transition temperature, and thermal stability of the PU foams increased. The results in this study indicated the microwave liquefied bagasse products have potential for the fabrication of polyurethane foams.
